# Supplementary material for: Impact of screening on cervical cancer incidence in England: a time trend analysis
Source: BMJ Open. 2019 Jan 24;9(1):e026292. doi: 10.1136/bmjopen-2018-026292 (PMC6347909; doi:10.1136/bmjopen-2018-026292)
Supplement: Supplementary file 1 [file bmjopen-2018-026292supp001.pdf]

Supplementary materials for “The impact of screening on cervical cancer incidence in England: a time trend analysis”

Supplementary 1

Supplementary Table 1. Model fit for data 1971 to 1987 using the log link function

		Log link		Power Link	
	**df**	**AIC**	**Pseudo R^2**	**AIC**	**Pseudo R2**
Null		323.4	0	323.4	0
A	1063	22.9	.929	22.9	.929
Adrift (in cohort)	1062	22.8	.930	22.7	.930
AP	1060	22.1	0.932	21.9	.932
AC	1059	14.4	0.956	14.9	.954
APC	1056	14.2	0.956	14.6	.955
ACP*A	1041	11.9	0.963	12.3	.962

Supplementary 2

Validation analysis and HPV age adjustment selection

In our validation analyses to identify the best weeks/year adjustment to define biological age, we used observed data between 1971 and 2001 to predict incidence rates up to 2013 applying a number of weeks/year adjustments (i.e. x= 0 to 12) in our modified APC model. To quantify how well the model predicted incidence for years 2002 to 2013, we used a modified Pearson Chi-squared statistic:

$$(\text{estimated}-\text{observed})^2/(0.5+\text{observed}) \qquad (1)$$

where the observed value was used in the denominator as it is the best estimator of the variance of the observed count since, for projections, the ‘expected count’ may not reflect the true expectation. Small values indicate a good fit to the data.

Figure 1 plots the obtained Pearson’s Chi-squared against the various age adjustments. Our validation analyses showed that, overall, a 2- to 7-weeks/year adjustment offered a good fit to the data and greater adjustments (i.e. 5-7 weeks/year) fit the data best when projections were longer into the future (i.e. 6+ years).

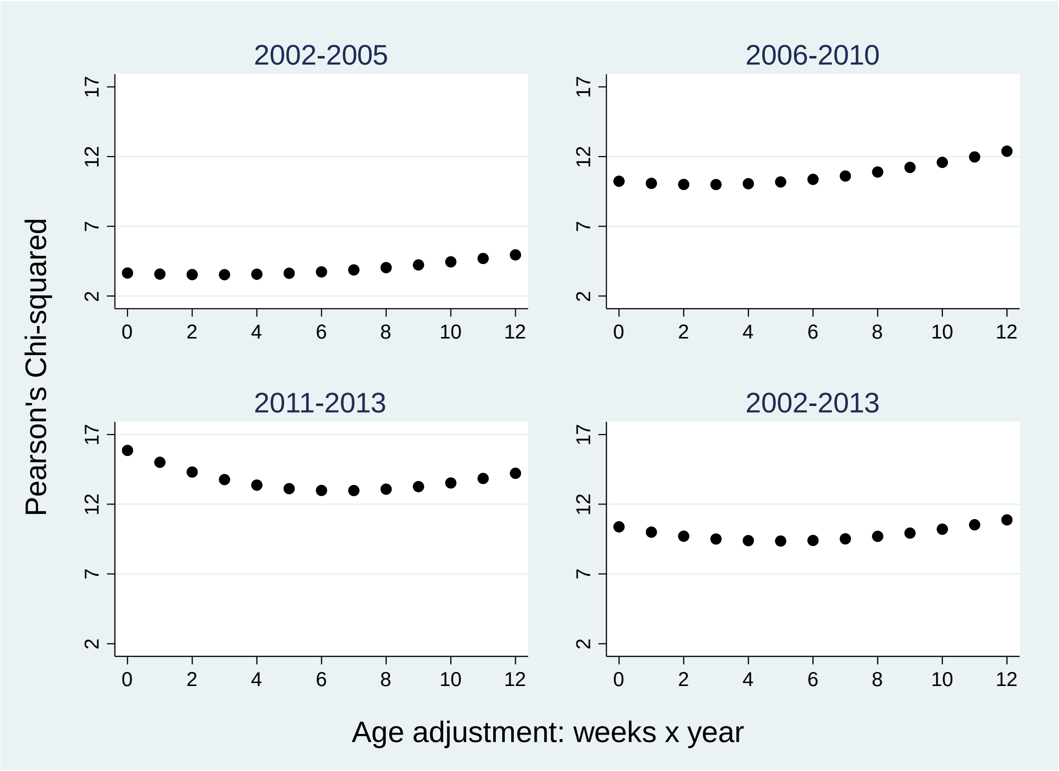

Figure S2. Goodness of fit indices for a number of weeks/year age adjustments (i.e. biological age) using observed data between 1971 and 2001 to predict incidence rates up to 2013.

Supplementary 3

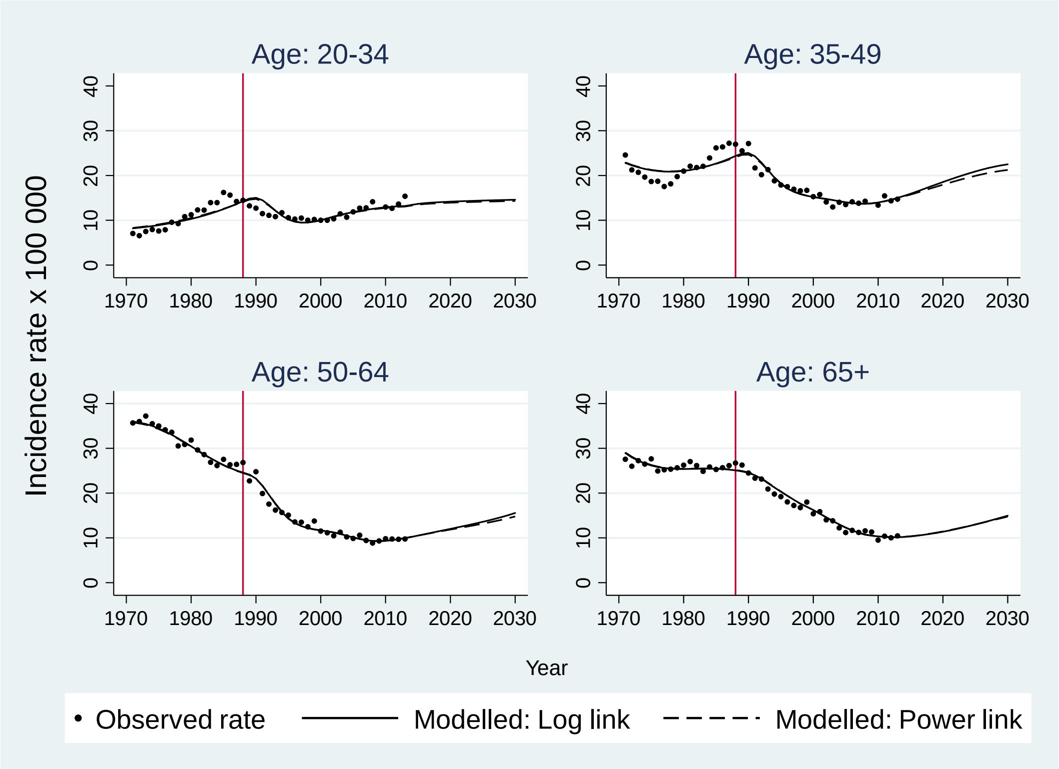

Figure S1. Cervical cancer incidence by age: Observed and estimated rates, using the log and 'power-5' link functions and a 5 weeks/year age adjustment (i.e. biological age)

Supplementary 4

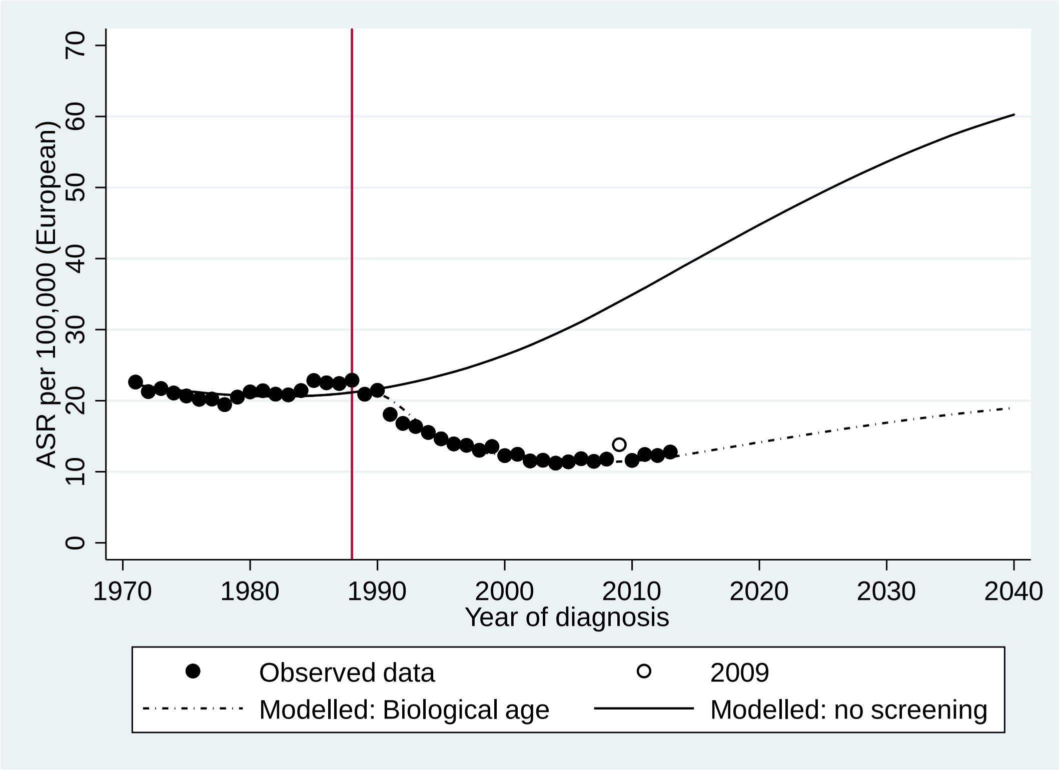

Figure S3. Age Standardised Rate (European population) in women aged 20 to 84. Cervical cancer incidence comparing observed data, projections under the current scenario and the no-screening scenario using a 5 weeks/year age adjustment (i.e. biological age).
