# Supplementary material for: Impact of screening on cervical cancer incidence in England: a time trend analysis
Source: BMJ Open. 2019 Jan 24;9(1):e026292. doi: 10.1136/bmjopen-2018-026292 (PMC6347909; doi:10.1136/bmjopen-2018-026292)
Supplement: Author's manuscript [file bmjopen-2018-026292.draft_revisions.pdf]

BMJ Open is committed to open peer review. As part of this commitment we make the peer review history of every article we publish publicly available.

When an article is published we post the peer reviewers' comments and the authors' responses online. We also post the versions of the paper that were used during peer review. These are the versions that the peer review comments apply to.

The versions of the paper that follow are the versions that were submitted during the peer review process. They are not the versions of record or the final published versions. They should not be cited or distributed as the published version of this manuscript.

BMJ Open is an open access journal and the full, final, typeset and author-corrected version of record of the manuscript is available on our site with no access controls, subscription charges or pay-per-view fees (http://bmjopen.bmj.com).

If you have any questions on BMJ Open's open peer review process please email editorial.bmjopen@bmj.com

BMJ Open

The impact of screening on cervical cancer incidence in England: a time trend analysis

Journal:	*BMJ Open*
Manuscript ID	bmjopen-2018-026292
Article Type:	Research
Date Submitted by the Author:	24-Aug-2018

Complete List of Authors:	Pesola, Francesca; King’s College London , School of Cancer and Pharmaceutical Sciences<br>Sasieni, Peter; Kings College London

Keywords:	PUBLIC HEALTH, Epidemiology < THORACIC MEDICINE, STATISTICS & RESEARCH METHODS, Adult oncology < ONCOLOGY

SCHOLARONE™
Manuscripts

For peer review only

The impact of screening on cervical cancer incidence in England: a time trend analysis

Francesca Pesola[1]* and Peter Sasieni[1]

King's College London, London, UK

School of Cancer and Pharmaceutical Sciences, Faculty of Life Sciences & Medicine, King's College London, Innovation Hub, Guy's Hospital, Great Maze Pond, London SE1 9RT, UK

* Corresponding author:

Francesca Pesola,

King's College London

Faculty of Life Sciences & Medicine

School of Cancer and Pharmaceutical Sciences

Innovation Hub, Guy's Hospital

Great Maze Pond

London SE1 9RT

francesca.pesola@kcl.ac.uk

Strengths and limitations of this study

For peer review only

ABSTRACT

Objective To better model underlying trends in cervical cancer incidence so as to model past trends, estimate the impact of cervical screening on cervical cancer rates at different ages, and to obtain a counterfactual baseline under a no-screening scenario.

Design Individual level, trend analysis of cancer registry data recorded between 1971 and 2013.

Setting England.

Participants 132 493 women aged 20-84 with a diagnosis of cervical cancer.

Outcome measure Cervical cancer incidence data were modelled using a modified age period cohort (APC) model, able to capture both increased exposure to human papillomavirus (HPV) as well as changes in the age of exposure to HPV in young cohorts. Observed rates were compared with counterfactual baseline rates, estimated under a no-screening scenario, to estimate the protective effect of screening.

Results Rates of cervical cancer incidence have been decreasing since the introduction of the screening programme in 1988 but are estimated to increase in the future under the current scenario. We estimate that the age of first HPV exposure has decreased by about 1 year every decade since the early 1970s (women born in 1955 onwards). Between 1988 and 2013, it was estimated that screening had prevented over 65000. Moreover, in 2013, the age-standardised rate (ASR) estimated under the no-screening scenario (26.5, 95%CIs: 25.4 to 27.6) was threefold the observed ASR (8.2, 95%CIs: 7.9 to 8.6) among women aged 20-84.

Conclusions Our results showed the importance of screening in preventing cervical cancer and indicated future rates are dependent on age at HPV exposure. Estimated future rates can be used for healthcare planning while the counterfactual baseline to quantify the impact of HPV vaccination in microsimulations.

Keywords: Age-Period-Cohort model; cervical cancer, incidence rates, past trends, screening, HPV exposure, prediction.

For peer review only - http://bmjopen.bmj.com/site/about/guidelines.xhtml

Strengths and limitations of this study

- Individual level data for cervical cancer incidence over a 40-year window.
- First study to use a modified age period cohort (APC) model able to capture generational changes in the prevalence of human papillomavirus (HPV) as well as changes in age at first exposure.
- The model allows researchers and policy makers to estimate the effectiveness of screening as well as the effect of HPV vaccination and screening in the future by obtaining a counterfactual baseline.
- Our revised model could be used to study other diseases where age at exposure and intensity of exposure affect future age-specific incidence (e.g., lung cancer).
- One limitation, typical of APC-based analyses, is that estimates are dependent on the assumptions of the model.

For peer review only

INTRODUCTION

It is essential to be able to estimate the future burden of cervical cancer to ensure sufficient resources and services are in place to deal with demands; however, this task is complicated because different risk and preventive factors affect incidence rates of cervical cancer. Specifically, national screening programmes are a main protective factor together with HPV vaccination. [1-3] Indeed, in England the incidence of cervical cancer has decreased by over a third (from 15.0 to 9.8 per 100,000) since the introduction of the national cervical screening programme in 1988; however, this reduction has slowed in recent years and incidence rates in women under the age of 30 have been increasing. [4] This increase may be driven by greater and earlier exposure to persistent infection of human papillomavirus (HPV), which has been identified as the main risk factor for cervical cancer. [5,6]

To estimate the future burden of cancer, epidemiologists and medical researchers have implemented age period cohort (APC) models. When exploring trends of cervical cancer, the period effect is a proxy for the effect of screening while the cohort effect captures generational changes to HPV exposure and, specifically, increased intensity (i.e. incidence) in younger cohorts. [7-9] In a recent study, past incidence rates of cervical cancer in the UK were extrapolated to 2035 using an APC model. [10] The overall age-standardised rate (ASR) was predicted to increase by 43% between 2014 and 2035. This increase was observed especially amongst women aged 25 to 49, for whom estimated rates were predicted to be higher by 2035 than they were prior to the introduction of screening. The epidemic predicted in this study is driven by high rates of cervical cancer observed in young women in recent years. Indeed, a standard APC model assumes younger cohorts will have very high rates of cancer throughout their lives due to their high risk at age 20-29 as this high risk is expected to apply to all ages. We suggest that the timing of the exposure is also important since the risk of cervical cancer was found to be a function of age at first HPV exposure. [11] Hence, the cohort effect needs to capture increased incidence as well as earlier HPV exposure amongst younger women. Earlier HPV exposure explains the increased rates of cervical cancer in younger women but its impact will be minimal by the time these women reach their 40s, 50s, and 60s, as the timing of first exposure becomes less relevant over time. The assumption that earlier HPV exposure is cohort dependent is supported by evidence which shows earlier age at intercourse, a proxy for HPV exposure, in younger cohorts. [12,13]

To explore past trends and estimate future incidence rates of cervical cancer, we developed a modified APC model implicitly able to capture birth-cohort changes in HPV exposure both in terms of changes to cumulative exposure (by age 35) and changes to the age at which women are infected. The latter is the innovative feature of our approach. An important output of our model is that it also allows us to estimate rates under a scenario where no screening is available (i.e. counterfactual baseline). This

For peer review only - http://bmjopen.bmj.com/site/about/guidelines.xhtml

counterfactual baseline can be used to quantify the protective effect of screening and the effect of HPV vaccination and screening in future cohorts. [14 15]

METHODS

Cases

Data on 132 493 (incidence of) cervical cancers in women aged 20-84 diagnosed between 1971 and 2013 in England were provided by Public Health England. Data were broken down by year of diagnosis and single year of age. The population estimates (women aged 20-84) for 1971-2014 and projections to 2030 were obtained from the Office for National Statistics.[16]

Cancers diagnosed in women aged 24- 25 in the most recent years (2010-2013) were excluded from our analyses as they tend to be early stage cancers (FIGO stage IA) and reflect prevalent disease detected at the first screen. [17 18] Thus, they would require separate modelling. Cancers diagnosed in 2009 for women aged 20 to 49 were also excluded, as there was a substantial one-off increase in diagnostic screening and symptomatic testing in 2009 as a result of Jade Goody's cancer diagnosis and death. [19 20]

APC model

APC models, assuming a Poisson distribution of cancer events, are described by the following formula:

$$\log(\lambda(\text{age, period})) = f_A(\text{age}) + f_C(\text{cohort}) + f_P(\text{period}) \qquad (1)$$

where λ is the incidence rate, and f_A, f_p and f_C are functions of (chronological) age, period (i.e. year of incidence) and cohort (i.e. year of birth approximated by year of diagnosis minus age at diagnosis), respectively. These functions are taken to be natural cubic splines as they offer greater flexibility and allow us to model more realistic trends and projections than models which use the step function since change occurs smoothly rather than in sudden jumps.

The period effect is a proxy of the effect of screening, which was set to be 0 prior to the introduction of the screening programme (1988) and women born before 1924, as these birth cohorts would have not benefited from the protective effect of screening when the programme was first launched.

The impact of screening has been found to differ by age group as screening is more effective at older ages [21] (Sasieni *et al*, 2009) and wanes after last screen [22]. Hence, we modelled the impact of

For peer review only - http://bmjopen.bmj.com/site/about/guidelines.xhtml

screening separately for women aged 20-34, 45-49, 50-64, 65-69, 70-74 and 75-84. To capture the effect of screening in different age groups, our modified model (i.e. ACP*A) was defined as follows:

$$\log(\lambda(\text{age, period}))=\log(\lambda(\text{age, period})) = f_A(\text{b\_age}) + f_C(\text{cohort}) + f_{P,a\text{-}b}(\text{period,age}) \quad (2)$$

where $f_{P,a\text{-}b}$ is a smooth function of year of diagnosis, for each age-group (a-b), forced to be zero for cohorts not invited to screening ($f_{P,a\text{-}b}$(period,age)=0 if period≤1988 and cohort<1924), while b_age is the biological age which was defined using the following formula:

$$\text{b\_age} = \text{age}+\max(0, \text{year of birth}-1955)*x \quad (3)$$

where x represents the number of week(s) per cohort year (i.e. weeks/year; i.e. age adjustment) to be added to each birth cohort after 1955 and 'age' is the chronological age in years.

Social changes and the introduction of oral contraceptive in the 1960s led to changes in sexual behaviour (e.g. younger age at first intercourse and greater number of sexual partners) and, subsequently, a rise in sexually transmitted diseases in the 1970s. [23] We assume that women who were teenagers in the 1970s have greater and earlier HPV exposure than women from previous cohorts. Thus, in our model, we assumed women born after 1955 (i.e. aged 15 in 1970) have been exposed to HPV for x weeks/year longer than women born before 1955. For example, using a 5-weeks/year adjustment, by 2001 women born in 1975 (chronological age = 26) are expected to have been exposed to HPV for approximately 2 additional years and, therefore, have a risk equivalent to women aged 28 born before 1955. Thus, we model earlier exposure as having a risk equivalent to a woman from a pre-1955 cohort.

We compared our modified model (ACP*A) with simpler ones (vs. A, Adrift, AP, AC and standard APC) to identify the best model. We used the pseudo R-squared and deviance statistics rather than formal significance testing because with over 100 000 events, even very small changes in rates may be statistically significant. We conducted a series of validation analyses to identify the age adjustment which best approximated the data, in line with previous work exploring cancer incidence in the Nordic countries. [24] Using the best fitting model (i.e. ACP*A), the validation analyses were based on the observed data from 1971 to 2001 to predict incidence rates up to 2013 with several weeks/year age adjustments (i.e. 0 to 12). To identify the best age adjustment, we used a modified Pearson chi-squared statistic (see Appendix 1) to assess how close the projected values were to the observed ones across the estimated years (i.e. 2002-2013).

For peer review only - http://bmjopen.bmj.com/site/about/guidelines.xhtml

In all models, we fixed the future period effect to be the same as the one in the last year of observed data (i.e. 2013) and geometric damping was applied to birth cohorts after 1981. This was done as we are not expecting past trends to continue into the future and we are unsure how changes in sexual behaviour and HPV vaccination will affect future birth cohorts. Analyses were carried out in Stata 15.

Patient and public involvement

No patients or public were involved in the design or conduct of this study.

RESULTS

Model fit indices showed that our modified model (model VI) offered the best fit to the data (Table 1). The validation analyses showed a 2-7 weeks/year age adjustment offered a good fit to the data with greater (i.e. 5-7 weeks/year) fitting the data best when projections were longer into the future (i.e. 6+ years; Appendix 1). Thus, we used 5weeks/year for our projections.

Table 1. Model fit indices.

		df	Deviance	Pseudo R^2
Model I	A	2668	20284.8	0
Model II	A+drift (in coh)	2667	11846.5	0.3603
Model III	A+P (no year)	2664	10287.0	0.5135
Model IV	A+C	2661	6868.3	0.6039
Model V	A+C+P	2657	4977.9	0.6382
Model VI	A+C+A*P	2637	3974.5	0.6566

Figure 1 shows the observed date (dots) together with projections using chronological age (short-dash dot) compared to biological age with several adjustments. The greyed area shows the potential range of estimated rates based on different biological age adjustments (i.e. 2-7 weeks/year), with higher rates associated with a 2 week/year adjustment and lower rates obtained using a 7 weeks/year adjustment. The solid line is for the selected 5 weeks/year adjustment. The model provided a good fit to the observed data using either biological or chronological age; however, future projections differ depending on the age adjustment used. The model which fails to account for earlier exposure to HPV (short-dash-dot line) estimates future rates (2014-2030) higher than those observed in 1988. This is particularly true for women aged 35-64.

Using a 5 weeks/year adjustment, we calculated age-standardised rates (ASR; European standard population) for observed rates in 1972, 1992, 2012 and estimated rates 2032 as 3-year averages for all

For peer review only - http://bmjopen.bmj.com/site/about/guidelines.xhtml

ages combined; for example, rates for 2012 were obtained by averaging rates between 2011 and 2013. We found rates had dropped from 15 to 12 per 100 000 between 1972 and 1992 (-22% percentage change) and had further decrease to 9 per 100 000 in 2012 (percentage change -27%); however, our projections showed rates are expected to increase to 12% from 2012 to 2032 (+39% percentage change (ASR across all ages are presented in Appendix 2).

FIGURE1

The figure also depicts rates estimated under "no-screening" (i.e. a counterfactual baseline). These estimates were obtained by only considering the age and cohort effect (f_A and f_C) using a 5-weeks/year age adjustment (long-dash line). By 2013, over 65 000 cases (50%) of the expected cancers had been prevented since the introduction of screening in 1988. Additionally, results showed that, under the no-screening scenario, ASR would have been 3-fold higher in 2013; observed ASR 8.2 (95%Cis: 7.9 to 8.6) vs. no-screening scenario 26.5 (95%Cis: 25.4 to 27.6). We also find this would still be the case in 2030 assuming the current scenario continued. In 2013, rates in the no-screening scenario were higher across all age groups. The effect of screening appears greatest in the 50-64 age group, where rates would be nearly 4-fold higher under no screening (ASR: 1.8 (95%CIs: 1.6 to 1.9) vs. 6.9 (95%CIs: 6.3 to 7.5).

Screening effect

We explored the effect of screening (i.e. period effects) across different age groups. Figure 2 shows the relative risks (RRs) associated with the period effect for each age-group plotted over year of diagnosis. The figure shows RRs obtained using chronological age compared to biological age with a 5 weeks/year adjustment. The biological age adjustment does not affect results for women aged 50+ based on the model assumptions. Overall, screening reduces the relative risk associated with cancer incidence. This protective effect is more pronounced in women aged 35-64 (vs. <35) but it can still be observed amongst women aged 65-74 (and even 75-84), although steadily waning, as shown in Figure 2.

FIGURE2

DISCUSSION

Our modified APC model successfully captured past trends and efficiently estimated future incidence rates. Our study confirms the effectiveness of screening at reducing cervical cancer incidence across all ages. [7-9] The model showed the effect of screening was greater amongst women aged 35-64 (vs. <35), potentially as they have attended several screening rounds. Moreover, it captured the protective effect of screening in older women (65+). Indeed, we observed a waning protective effect which is

For peer review only - http://bmjopen.bmj.com/site/about/guidelines.xhtml

still considerable at age 74. This is in line with previous results by Castañon and colleagues [22] based on individual exposure to screening (rather than trends). The data suggest there is an effect of screening among women aged 65-69 in the first years after the introduction of the screening program. This observation is supported by data which indicate women in this age group were undergoing (opportunistic) screening. [25]

We quantified the effectiveness of the screening programme by comparing observed rates with rates estimated under a no-screening scenario. Results showed that, had screening not been introduced, overall incidence rates would be 3-fold higher, which is in line with previous results.[26] Overall, our results confirm findings from previous cohort and case-control studies while using population data, which are readily available.

In spite of the protective effect of screening, rates of cervical cancer are projected to increase over time, especially amongst women aged 35-64, potentially due to increased incidence of HPV infection and falling screening attendance. [27] These results should, however, be interpreted with caution as they rely on the assumption that the current scenario will continue; in reality, we are expecting HPV vaccination and the imminent introduction of HPV screening to have a further protective effect on women. [14 27] The counterfactual baseline obtained under the no-screening scenario can be used to quantify the effect of vaccination and HPV screening in the future.

Our results indicate the importance of capturing earlier and prolonged HPV exposure, together with changes in exposure intensity, when estimating future cervical cancer incidence. The assumption that women are exposure to HPV at an earlier age in more recent cohorts is supported by changes in sexual behaviour (e.g. earlier age at first intercourse) captured by data from NATSAL-3. [13] Failing to account for changes in age of first exposure to HPV leads to unrealistic future rates, which are in line with those observed in countries where screening is not systematically offered. [9] This is particularly noticeable amongst women aged 35-64 rather than younger women (<35). The impact of earlier exposure is minimal amongst women under the age of 35 due to the lag between HPV exposure and cancer. In a recent study, predicted cervical cancer rates for 2035 were higher than prior to 1988, especially amongst women aged 35 to 64. [10] These results may be due to the use of a standard APC model, which only captures increased intensity of exposure that is expected to last throughout a woman's lifetime. Moreover, we noticed the standard APC model failed to capture the differential effect of screening by age group. Indeed, using chronological age in our data, screening appeared to have a greater protective effect amongst younger women (<35) than those aged 35-64. This is counter to previous evidence which found screening to be more effective in older women (40+ vs. <40). [28] These observations further support the need to consider cohort effects that account for changes in age

For peer review only - http://bmjopen.bmj.com/site/about/guidelines.xhtml

at first exposure in addition to changes in the proportion of the population exposed, already captured by conventional APC models.

The main strength of our analysis is the use of individual level data over a 40-year window which permit predictions using modelling; however, there are some limitations. A typical limitation of APC analysis is that predicted rates are dependent on the model's assumption but our validation analyses suggest our assumptions are valid under the current scenario. Additionally, our study does not model the effect of HPV vaccination in younger cohorts, offered in England since 2008. However, the counterfactual no-screening scenario baseline from our model, was used in a microsimulation study to evaluate the appropriate screening intensity in HPV vaccinated and unvaccinated women. [15] This counterfactual baseline can also be used as a reference to quantify the effect of HPV vaccination and screening in the future. Our incidence rates are calculated for the overall population and we did not exclude women who have undergone hysterectomy from the denominator. However, in England and Wales hysterectomy prevalence is low, with a rate of 10.3% amongst women aged 25-64 (1991-995), and the extrapolated hysterectomy prevalence appears stable up to 2030. [29] Therefore, we are confident our estimated trends closely mirror the trends in cancer incidence in women with a cervix, even though, we acknowledge, the rates in older women with a cervix will be somewhat higher than the rates in all older women.

Conclusions

Using a modified APC model on cancer registry data, we confirmed the protective effect of screening with this effect lasting for over 10 years after the last screening invitation. Our results support the epidemiology of cervical cancer in younger cohorts being affected by earlier onset of sexual behavour, as reported in previous studies, [13 23] and not just the greater number of sexual partners. We found that failing to account for changes in age of first HPV exposure leads to unrealistic future estimates and, therefore, our modified approach should be used when modelling indicidence of cervical cancer. Moreover, the use of our model is not restricted to cervical cancer but could be implemebted by researchers studying other diseases (e.g., lung cancer) where age at exposure, as well as intensity of exposure, may determine future age-specific incidence.

For peer review only - http://bmjopen.bmj.com/site/about/guidelines.xhtml

REFERENCES

1. Andrae B, Kemetli L, Sparen P, et al. Screening-preventable cervical cancer risks: evidence from a nationwide audit in Sweden. *Journal of the National Cancer Institute* 2008;100(9):622-9. doi: 10.1093/jnci/djn099 [published Online First: 2008/05/01]
2. IARC Working Group on Cervical Cancer Screening. Efficacy of Screening chapter. In: Hakama M MA, Day NE, eds, ed. Screening for Cancer of the Uterine Cervix IARC Scientific Publications N 76. Lyon: IARC1986.
3. Lowy DR. HPV vaccination to prevent cervical cancer and other HPV-associated disease: from basic science to effective interventions. *The Journal of Clinical Investigation* 2016;126(1):5-11. doi: 10.1172/JCI85446
4. Foley G, Alston R, Geraci M, et al. Increasing rates of cervical cancer in young women in England: an analysis of national data 1982-2006. *Br J Cancer* 2011;105(1):177-84. doi: 10.1038/bjc.2011.196 [published Online First: 2011/06/10]
5. Cuzick J, Sasieni P, Davies P, et al. A systematic review of the role of human papilloma virus (HPV) testing within a cervical screening programme: summary and conclusions. *Br J Cancer* 2000;83(5):561-5. doi: 10.1054/bjoc.2000.1375 [published Online First: 2000/08/17]
6. Walboomers JM, Jacobs MV, Manos MM, et al. Human papillomavirus is a necessary cause of invasive cervical cancer worldwide. *The Journal of pathology* 1999;189(1):12-9. doi: 10.1002/(sici)1096-9896(199909)189:1<12::aid-path431>3.0.co;2-f [published Online First: 1999/08/19]
7. Sasieni P, Adams J. Analysis of cervical cancer mortality and incidence data from England and Wales: evidence of a beneficial effect of screening. *Journal of the Royal Statistical Society: Series A (Statistics in Society)* 2000;163(2):191-209. doi: 10.1111/1467-985X.00165
8. Vaccarella S, Franceschi S, Engholm G, et al. 50 years of screening in the Nordic countries: quantifying the effects on cervical cancer incidence. *Br J Cancer* 2014;111(5):965-69. doi: 10.1038/bjc.2014.362
9. Vaccarella S, Franceschi S, Zaridze D, et al. Preventable fractions of cervical cancer via effective screening in six Baltic, central, and eastern European countries 2017-40: a population-based study. *The Lancet Oncology* 2016;17(10):1445-52. doi: 10.1016/s1470-2045(16)30275-3 [published Online First: 2016/08/28]
10. Smittenaar CR, Petersen KA, Stewart K, et al. Cancer incidence and mortality projections in the UK until 2035. *Br J Cancer* 2016;115(9):1147-55. doi: 10.1038/bjc.2016.304 [published Online First: 2016/10/26]
11. Plummer M, Peto J, Franceschi S. Time since first sexual intercourse and the risk of cervical cancer. *International journal of cancer* 2012;130(11):2638-44. doi: 10.1002/ijc.26250 [published Online First: 2011/06/28]
12. Cuzick J, Sasieni P, Singer A. Risk factors for invasive cervix cancer in young women. *European journal of cancer (Oxford, England : 1990)* 1996;32a(5):836-41. [published Online First: 1996/05/01]
13. Mercer CH, Tanton C, Prah P, et al. Changes in sexual attitudes and lifestyles in Britain through the life course and over time: findings from the National Surveys of Sexual Attitudes and Lifestyles (Natsal). *Lancet (London, England)* 2013;382(9907):1781-94. doi: 10.1016/s0140-6736(13)62035-8 [published Online First: 2013/11/30]
14. Castanon A, Landy R, Sasieni P. By how much could screening by primary human papillomavirus testing reduce cervical cancer incidence in England? *Journal of medical screening* 2017;24(2):110-12. doi: 10.1177/0969141316654197 [published Online First: 2016/07/02]
15. Landy R, Windridge P, Gillman MS, et al. What cervical screening is appropriate for women who have been vaccinated against high risk HPV? A simulation study. *International journal of cancer* 2018;142(4):709-18. doi: 10.1002/ijc.31094 [published Online First: 2017/10/13]
16. ONS. Mid-1971 to Mid-2013 Population Estimates for UK, constituent countries and English regions, 2014.

For peer review only - http://bmjopen.bmj.com/site/about/guidelines.xhtml

17. Castanon A, Leung VMW, Landy R, et al. Characteristics and screening history of women diagnosed with cervical cancer aged 20-29 years. *Br J Cancer* 2013;109(1):35-41. doi: 10.1038/bjc.2013.322
18. Castanon A, Sasieni P. Is the recent increase in cervical cancer in women aged 20-24years in England a cause for concern? *Preventive medicine* 2018;107:21-28. doi: 10.1016/j.ypmed.2017.12.002 [published Online First: 2017/12/17]
19. Lancucki L, Sasieni P, Patnick J, et al. The impact of Jade Goody's diagnosis and death on the NHS Cervical Screening Programme. *Journal of medical screening* 2012;19(2):89-93. doi: 10.1258/jms.2012.012028 [published Online First: 2012/06/02]
20. Sasieni P, Castanon A. Dramatic increase in cervical cancer registrations in young women in 2009 in England unlikely to be due to the new policy not to screen women aged 20-24. *Journal of medical screening* 2012;19(3):127-32. doi: 10.1258/jms.2012.012081 [published Online First: 2012/10/25]
21. Sasieni P, Castanon A, Cuzick J. Effectiveness of cervical screening with age: population based case-control study of prospectively recorded data. *BMJ (Clinical research ed)* 2009;339:b2968. doi: 10.1136/bmj.b2968 [published Online First: 2009/07/30]
22. Castanon A, Landy R, Cuzick J, et al. Cervical screening at age 50-64 years and the risk of cervical cancer at age 65 years and older: population-based case control study. *PLoS medicine* 2014;11(1):e1001585. doi: 10.1371/journal.pmed.1001585 [published Online First: 2014/01/24]
23. Simms I, Fairley CK. Epidemiology of genital warts in England and Wales: 1971 to 1994. *Genitourinary medicine* 1997;73(5):365-7. [published Online First: 1998/04/16]
24. Moller B, Fekjaer H, Hakulinen T, et al. Prediction of cancer incidence in the Nordic countries: empirical comparison of different approaches. *Stat Med* 2003;22(17):2751-66. doi: 10.1002/sim.1481 [published Online First: 2003/08/27]
25. DoH. Cervical Cytology: Summary Information From Form KC53. London, United Kingdom: Department of Health Statistics Division, 1989-90.
26. Landy R, Pesola F, Castanon A, et al. Impact of cervical screening on cervical cancer mortality: estimation using stage-specific results from a nested case-control study. *Br J Cancer* 2016;115(9):1140-46. doi: 10.1038/bjc.2016.290 [published Online First: 2016/10/26]
27. Castanon A, Landy R, Pesola F, et al. Prediction of cervical cancer incidence in England, UK, up to 2040, under four scenarios: a modelling study. *The Lancet Public health* 2018;3(1):e34-e43. doi: 10.1016/s2468-2667(17)30222-0 [published Online First: 2018/01/09]
28. Sasieni P, Adams J, Cuzick J. Benefit of cervical screening at different ages: evidence from the UK audit of screening histories. *Br J Cancer* 2003;89(1):88-93. doi: 10.1038/sj.bjc.6600974 [published Online First: 2003/07/03]
29. Redburn JC, Murphy MF. Hysterectomy prevalence and adjusted cervical and uterine cancer rates in England and Wales. *BJOG : an international journal of obstetrics and gynaecology* 2001;108(4):388-95. [published Online First: 2001/04/18]

For peer review only - http://bmjopen.bmj.com/site/about/guidelines.xhtml

CAPTIONS

Figure 1. Trends and projections based on our modified APC model across different age groups. The solid circles represent the observed data. Estimated rates for the observed period are for chronological age (short-dash dot) and biological age using a 5 week/year adjustment (solid line). The greyed area shows the potential range of estimated future rates based on the chronological age using 2 and 7 week/year age adjustments (biological age). The rates estimated under the no-screening scenario are represented by the long-dash dot line.

Figure 2. Relative Risk (RR) of the period effect showing the effect of screening by age group for a) chronological age (solid circle), and b) biological age using a 5-week/year age adjustment (hollow circle).

For peer review only

For peer review only - http://bmjopen.bmj.com/site/about/guidelines.xhtml

Funding: The research was supported by a grant from Cancer Research UK, C8162/A16892.

Competing interests: None

Authors' contributions: FP was responsible for study design, data analysis and drafted the paper. PS was responsible for study concept and design and contributed to editing of the paper.

Data sharing statement: The data are available and can be obtained from Public Health England through the Office for Data Release (ODR). The authors can provide data specification for the data request

For peer review only

For peer review only - http://bmjopen.bmj.com/site/about/guidelines.xhtml

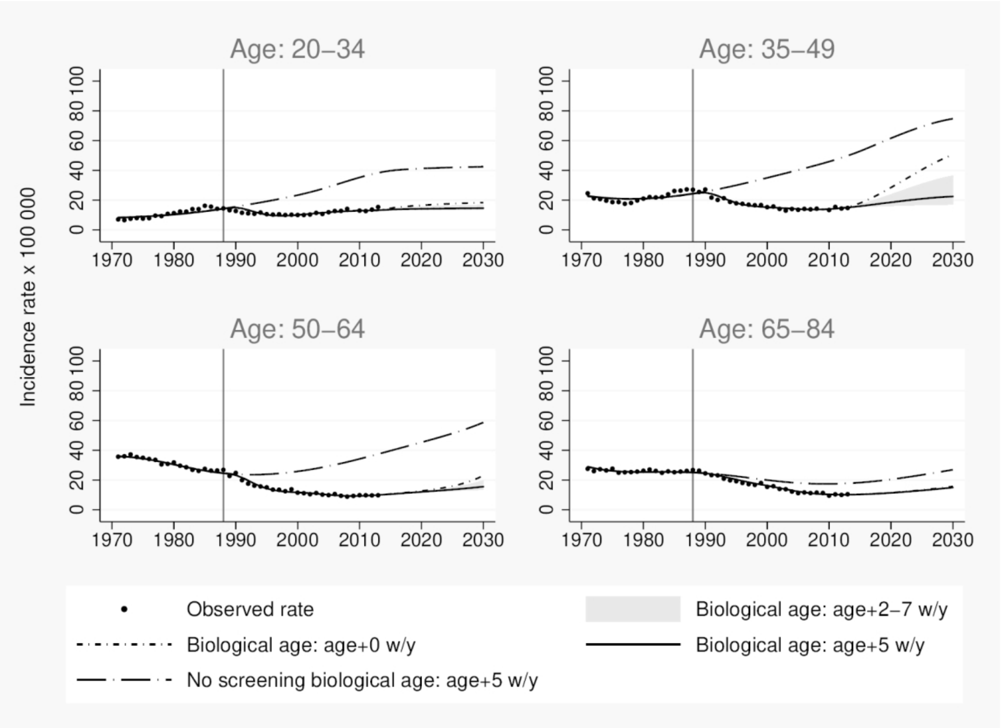

Figure 1. Trends and projections based on our modified APC model across different age groups. The solid circles represent the observed data. Estimated rates for the observed period are for chronological age (short-dash dot) and biological age using a 5 week/year adjustment (solid line). The greyed area shows the potential range of estimated future rates based on the chronological age using 2 and 7 week/year age adjustments (biological age). The rates estimated under the no-screening scenario are represented by the long-dash dot line.

139x101mm (300 x 300 DPI)

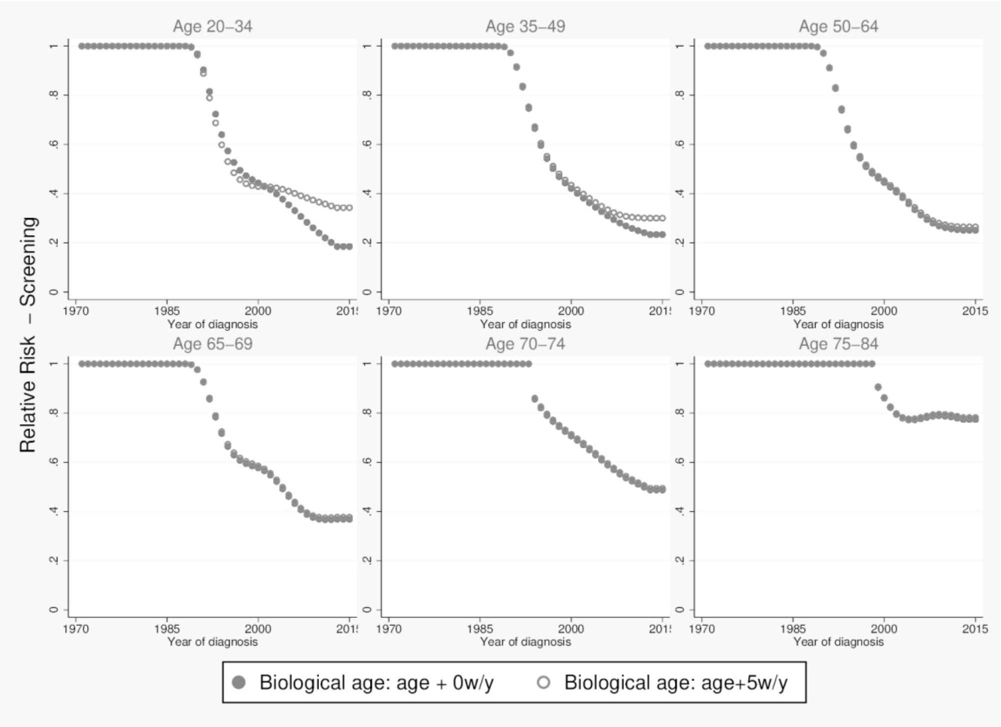

Figure 2. Relative Risk (RR) of the period effect showing the effect of screening by age group for a) chronological age (solid circle), and b) biological age using a 5-week/year age adjustment (hollow circle).

139x101mm (300 x 300 DPI)

Supplementary materials for “The impact of screening on cervical cancer incidence in England: a time trend analysis”

Supplementary 1 - Validation analysis and HPV age adjustment selection

In our validation analyses, observed data between 1971 and 2001 was used to predict incidence rates up to 2013 applying a number of week/year adjustments (i.e. 0 to 12) using our modified APC model. We used a modified Pearson’s Chi-squared statistics To quantify how well the model predicted incidence for years 2002 to 2013, we used a modified Pearson chi-squared statistic:

(estimated-observed)^2/(0.5+observed) (1)

where the observed value was used in the denominator as it is the best estimator of the variance of the observed count since, for projections, the ‘expected count’ may not reflect the true expectation. Small values indicate a good fit to the data.

Figure 1 plots the obtained Pearson’s chi-squared against the various age adjustments. Our validation analyses showed that, overall, a 2- to 7-weeks/year adjustment offered a good fit to the data and greater adjustments (i.e. 5-7 weeks/year) fit the data best when projections were longer into the future (i.e. 6+ years).

For peer review only

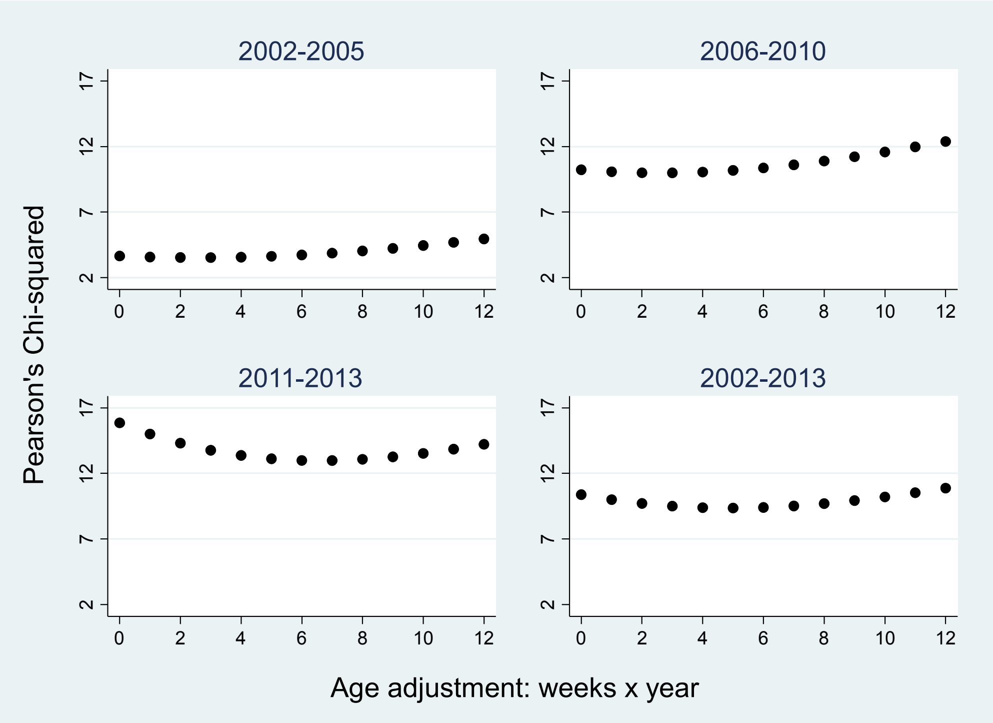

Figure S1. Goodness of fit indices for a number of week per year adjustments for age using observed data between 1971 and 2001 to predict incidence rates up to 2013.

For peer review only

Supplementary 2 - Age-Standardised rates (ASR) figure

ASR for observed rates vs. estimated rates using a no-screening scenario using a 5 weeks/year age adjustment.

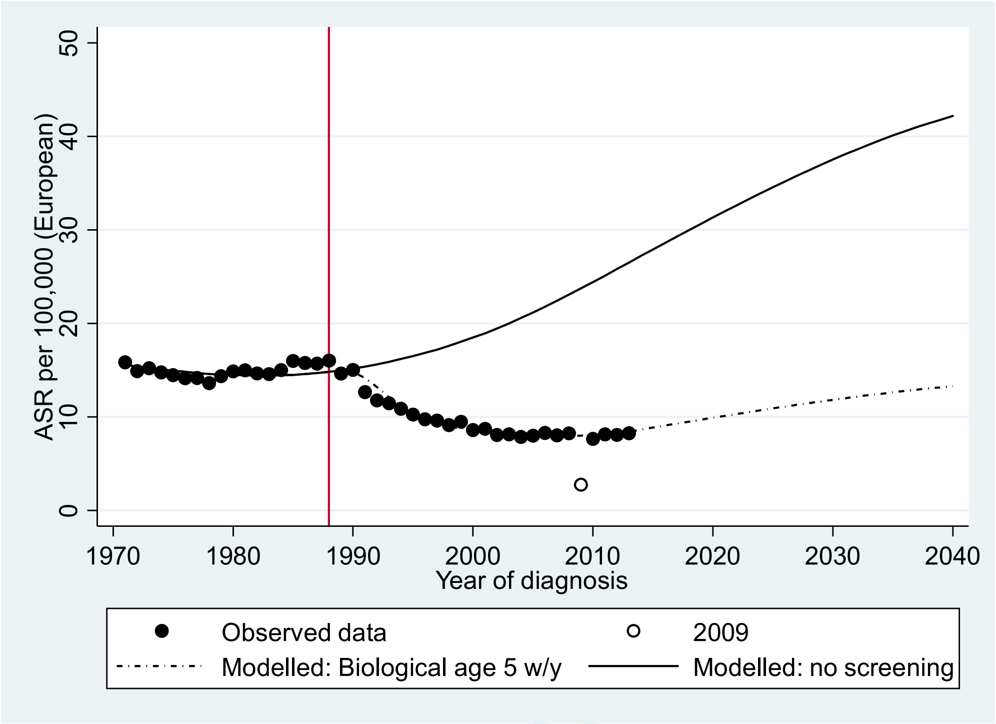

Figure S2. Age Standardised Rate (European population) of cervical cancer incidence comparing observed data vs. the no-screening scenario using a 5-year age adjustment.

BMJ Open

The impact of screening on cervical cancer incidence in England: a time trend analysis

Journal:	*BMJ Open*
Manuscript ID	bmjopen-2018-026292.R1
Article Type:	Research
Date Submitted by the Author:	16-Nov-2018
Complete List of Authors:	Pesola, Francesca; King's College London , School of Cancer and Pharmaceutical Sciences<br>Sasieni, Peter; Kings College London
**Primary Subject Heading**:	Epidemiology
Secondary Subject Heading:	Oncology
Keywords:	PUBLIC HEALTH, STATISTICS & RESEARCH METHODS, Adult oncology < ONCOLOGY

SCHOLARONE™
Manuscripts

The impact of screening on cervical cancer incidence in England: a time trend analysis

Francesca Pesola[1]* and Peter Sasieni[1]

King's College London, London, UK

School of Cancer and Pharmaceutical Sciences, Faculty of Life Sciences & Medicine, King's College London, Innovation Hub, Guy's Hospital, Great Maze Pond, London SE1 9RT, UK

* Corresponding author:

Francesca Pesola,

King's College London

Faculty of Life Sciences & Medicine

School of Cancer and Pharmaceutical Sciences

Innovation Hub, Guy's Hospital

Great Maze Pond

London SE1 9RT

francesca.pesola@kcl.ac.uk

For peer review only

For peer review only - http://bmjopen.bmj.com/site/about/guidelines.xhtml

ABSTRACT

Objective To better model underlying trends in cervical cancer incidence so as to model past trends, estimate the impact of cervical screening on cervical cancer rates at different ages, and to obtain a counterfactual baseline under a no-screening scenario.

Design Trend analysis of cancer registry data recorded between 1971 and 2013.

Setting England.

Participants 132 493 women aged 20-84 with a diagnosis of cervical cancer.

Outcome measure Cervical cancer incidence data were modelled using a modified age period cohort (APC) model, able to capture both increased exposure to human papillomavirus (HPV) as well as changes in the age of exposure to HPV in young cohorts. Observed rates were compared with counterfactual baseline rates under a no-screening scenario, to estimate the protective effect of screening.

Results Rates of cervical cancer incidence have been decreasing since the introduction of screening but are projected to increase in the future under the current scenario. Between 1988 and 2013, it was estimated that screening had prevented approximately 65000 cancers. Moreover, in 2013, the age-standardised rate (ASR) estimated under the no-screening scenario (37.9, 95%CIs: 37.4 to 38.3) was threefold higher among women aged 20-84 than the observed ASR (12.8, 95%CIs: 12.3 to 13.3). We estimate that the age of first HPV exposure has decreased by about 1 year every decade since the early 1970s (women born in 1955 onwards).

Conclusions Our results corroborated the importance of screening in preventing cervical cancer and indicated future rates are dependent on age at HPV exposure. Estimated future rates can be used for healthcare planning while the counterfactual baseline to quantify the impact of HPV vaccination in microsimulations.

Keywords: Age-Period-Cohort model; cervical cancer, incidence rates, past trends, screening, HPV exposure, prediction.

For peer review only

Strengths and limitations of this study

- Cervical cancer incidence over a 40-year period.
- First study to use a modified age period cohort (APC) model able to capture generational changes in the prevalence of human papillomavirus (HPV) as well as changes in age at first exposure.
- The model allows researchers and policy makers to estimate the effectiveness of screening as well as the effect of HPV vaccination and screening in the future by obtaining a counterfactual baseline.
- Our revised model could be used to study other diseases where age at exposure and intensity of exposure affect future age-specific incidence (e.g. lung cancer).
- One limitation, typical of APC-based analyses, is that estimates are dependent on the assumptions of the model.

For peer review only

For peer review only - http://bmjopen.bmj.com/site/about/guidelines.xhtml

INTRODUCTION

It is essential to be able to estimate the future burden of cervical cancer to ensure sufficient resources and services are in place to deal with demands; however, this task is complicated because different risk and preventive factors affect incidence rates of cervical cancer. Specifically, national screening programmes are a main protective factor together with HPV vaccination. [1-3] Indeed, in England the incidence of cervical cancer has decreased by over a third (from 15.0 to 9.8 per 100,000) since the introduction of the national cervical screening programme in 1988; however, this reduction has slowed in recent years and incidence rates in women under the age of 30 have been increasing. [4] This increase may be driven by greater and earlier exposure to persistent infection of human papillomavirus (HPV), which has been identified as the main risk factor for cervical cancer. [5-7]

To estimate the future burden of cancer, epidemiologists and medical researchers have implemented age period cohort (APC) models. When exploring trends of cervical cancer, the period effect is a proxy for the effect of screening while the cohort effect is interpreted as generational changes to HPV exposure and, specifically, increased intensity (i.e. incidence) in younger cohorts. [8-10] In a recent study, past incidence rates of cervical cancer in the UK were extrapolated to 2035 using an APC model. [11] The overall age-standardised rate (ASR) was predicted to increase by 43% between 2014 and 2035. This increase was observed especially amongst women aged 25 to 49, for whom estimated rates were predicted to be higher by 2035 than they were prior to the introduction of screening. The epidemic predicted in this study is driven by high rates of cervical cancer observed in young women in recent years. Indeed, a standard APC model assumes cohorts with very high rates of cervical cancer at ages 20-29 will continue to have high rates of cancer throughout their lives. We suggest that the timing of the exposure is also important since the risk of cervical cancer was found to be a function of age at first HPV exposure. [12] Hence, the cohort effect needs to capture increased as well as earlier HPV exposure amongst younger women. Earlier HPV exposure can explain the increased rates of cervical cancer in women in their 20s but its impact will be minimal by the time these women reach their 40s, 50s, and 60s. This is because cervical cancer incidence within a cohort increases sharply between age 20 and 29 but is relatively flat between age 40 and 60. The assumption that earlier HPV exposure is cohort dependent is supported by evidence which shows earlier age at intercourse, a proxy for HPV exposure, in younger cohorts. [13 14]

To explore past trends and estimate future incidence rates of cervical cancer, we developed a modified APC model implicitly able to capture birth-cohort changes in HPV exposure both in terms of changes to cumulative exposure (by age 35) and changes to the age at which women are infected. The latter is the innovative feature of our approach. An important output of our model is that it also allows us to estimate rates under a scenario where no screening is available (i.e. counterfactual baseline). This

For peer review only - http://bmjopen.bmj.com/site/about/guidelines.xhtml

counterfactual baseline can be used to quantify the protective effect of screening and the effect of HPV vaccination and screening in future cohorts. [15 16]

METHODS

Cases

Data on 132 493 (incidence of) cervical cancers in women aged 20-84 diagnosed between 1971 and 2013 in England were provided by Public Health England. Data were broken down by year of diagnosis and single year of age. The population estimates (women aged 20-84) for 1971-2014 and projections to 2030 were obtained from the Office for National Statistics.[17]

Cancers diagnosed in women aged 24- 25 in the most recent years (2010-2013) were excluded from our projections as they tend to be early stage cancers (FIGO stage IA) and reflect prevalent disease detected at the first screen. [18 19] Thus, they would require separate modelling. Cancers diagnosed in 2009 for women aged 20 to 49 were also excluded, as there was a substantial one-off increase in diagnostic screening and symptomatic testing in 2009 as a result of Jade Goody's cancer diagnosis and death. [20 21]

APC model

APC models, assuming a Poisson distribution of cancer events, are described by the following formula:

$$\lambda(\text{age, period}) = g^{-1}\left[f_A(\text{age}) + f_C(\text{cohort}) + f_P(\text{period})\right] \qquad (1)$$

where λ is the incidence rate as a function of age and calendar year, g is the link function, and f_A, f_p and f_C are functions of (chronological) age, period (i.e. year of incidence) and cohort (i.e. year of birth, approximated by year of diagnosis minus age at diagnosis), respectively. These functions are taken to be natural cubic splines as they offer greater flexibility and allow us to model more realistic trends and projections than models which use the step function since change occurs smoothly rather than in sudden jumps. The logarithmic and 'power-5' (i.e. $g(x) = x^{-5}$) link functions were used as both have been found to offer a good fit to the data. [22]

The period effect is a proxy of the effect of screening, which was set to be 0 prior to the introduction of the screening programme (1988) and for women born before 1924, as these birth cohorts would have not benefited from the protective effect of screening when the programme was first launched. The assumption of setting the period to 0 for years prior to 1988 was confirmed by preliminary analyses where we fitted the various models to data for years 1971 to 1987, included. The model

For peer review only - http://bmjopen.bmj.com/site/about/guidelines.xhtml

indices showed an age-cohort model offered a good fit to the data (Supplement 1). An age-cohort model was also shown to provide a good fit for cervical cancer incidence and mortality data pre-1988. [8]

The impact of screening has been found to differ by age group as screening is more effective at older ages [23-26] and wanes after the last screen [27]. Hence, we modelled screening separately for women aged 20-34, 45-49, 50-64, 65-69, 70-74 and 75-84. To capture the effect of screening in different age groups, our modified model (i.e. ACP*A) was defined as follows:

$$\lambda(\text{age, period}) = g^{-1}\,[f_A(\text{b\_age}) + f_C(\text{cohort}) + f_{P,a\text{-}b}(\text{period,age})] \qquad (2)$$

where $f_{P,a\text{-}b}$ is a smooth function of year of diagnosis, for each age-group (a-b), forced to be zero for cohorts not invited to screening ($f_{P,a\text{-}b}$(period,age)=0 if period≤1988 and cohort<1924), while b_age is the biological age which was defined using the following formula:

$$\text{b\_age} = \text{age}+\max(0, \text{year of birth}-1955)*x \qquad (3)$$

where x represents the number of week(s) per cohort year (i.e. weeks/year; i.e. age adjustment) to be added to each birth cohort after 1955 and 'age' is the chronological age in years. Therefore, the estimated biological age is a proxy for social changes in sexual behaviour and HPV exposure in successive generations post 1955.

Social changes and the introduction of oral contraceptive in the 1960s led to changes in sexual behaviour (e.g. younger age at first intercourse and greater number of sexual partners) and, subsequently, a rise in sexually transmitted diseases in the 1970s. [28] We assume that women who were teenagers in the 1970s (and afterwards) have greater and earlier HPV exposure than women from previous cohorts. Thus, in our model, we assumed women born after 1955 have been exposed to HPV for x weeks/year longer than women born before 1955. For example, using a 5 weeks/year adjustment, by 2001 women born in 1975 (chronological age = 26) are expected to have been exposed to HPV for approximately 2 additional years and, therefore, have a risk equivalent to women aged 28 born before 1955.

We compared our modified model (ACP*A) with simpler ones (vs. A, Adrift, AP, AC and standard APC) to identify the best model. We used the pseudo R-squared and Akaike information criterion (AIC) rather than formal significance testing because with over 100 000 events, even very small deviations from the model may be statistically significant. We conducted a series of validation analyses to identify the age adjustment which best approximated the data. Validation was done in line

For peer review only - http://bmjopen.bmj.com/site/about/guidelines.xhtml

with previous work exploring cancer incidence in the Nordic countries. [22] The validation used the observed data from 1971 to 2001 to predict incidence rates up to 2013. To identify the best age adjustment, we used a modified Pearson chi-squared statistic (Supplement 2) to assess how close the projected values were to the observed ones across the estimated years (i.e. 2002-2013) using several weeks/year age adjustments (i.e. x = 0 to 12) in equation 3.

In all models, we fixed the future period effect to be the same as the one in the last year of observed data (i.e. 2013). The cohort effect was allowed to change very slightly for those born after 1981 compared with the 1981 cohort. [29] This was done as we are not expecting past trends to continue into the future and we are unsure how changes in sexual behaviour and HPV vaccination will affect future birth cohorts. Analyses were carried out in Stata 15.

Patient and public involvement

No patients or public were involved in the design or conduct of this study. However, the request to model future cervical cancer rates came from Jo's Cervical Cancer Trust.

RESULTS

Model fit indices showed that our modified model (ACP*A) offers the best fit to the data using either link function (Table 1). Both the log and power link functions offered a good fit to the data; however, the results presented in the rest of the manuscript are based on the model using the log link function as it allows for clear interpretation of the relative risks associated with the separate effects. The conclusions did not differ depending on the link function used to model the data (Supplement 3).

Table 1. Model fit indices for the observed data using the log and power link functions

		**Log Link**		**Power link**	
	**df**	**AIC**	**Pseudo $R^{2\wedge}$**	**AIC**	**Pseudo $R^{2\wedge}$**
A	2668	99.4	0	99.1	0
Adrift (in cohort)	2667	44.1	0.557	46.0	0.536
AP	2664	37.0	0.628	39.6	0.600
AC	2661	21.0	0.789	20.5	0.793
APC	2657	14.1	0.856	14.5	0.854
ACP*A	2637	10.6	0.894	10.8	0.892

^ The age only model is treated as the null model to calculate the Pseudo R-squared

For peer review only - http://bmjopen.bmj.com/site/about/guidelines.xhtml

Moreover, using the best fitting model (i.e. ACP*A) the validation analyses showed a 2-7 weeks/year age adjustment offered a good fit to the data with greater adjustments (i.e. 5-7 weeks/year) fitting the data best when projections were longer into the future (i.e. 6+ years; Supplement 2). Thus, we used 5weeks/year for our projections.

Figure 1 shows the observed date (dots) together with projections using chronological age (short-dash dot) compared to biological age with several adjustments using the log function. The greyed area shows the potential range of estimated rates based on different biological age adjustments (i.e. 2-7 weeks/year), with higher rates associated with a 2 week/year adjustment and lower rates obtained using a 7 weeks/year adjustment. The solid line is for the selected 5 weeks/year adjustment. The model provided a good fit to the observed data using either biological or chronological age; however, future projections differ depending on the age adjustment used. The model, which failed to account for earlier exposure to HPV (short-dash-dot line), estimated future rates (2014-2030) higher than those observed in 1988. This was particularly true for women aged 35-64.

Using a 5 weeks/year adjustment, we calculated age-standardised rates (ASR; European standard population) for observed rates in 1972, 1992, 2012 and estimated rates 2032 as 3-year averages for all ages combined; for example, rates for 2012 were obtained by averaging rates between 2011 and 2013. We found the observed rates had dropped from 22 to 17 per 100 000 between 1972 and 1992 (-22% percentage change) and had further decreased to 13 per 100 000 in 2012 (percentage change -27%); however, our projections showed rates are expected to increase to 17% from 2012 to 2032 (+39% percentage change (ASR across all ages are presented in Supplement 4).

FIGURE1

The figure also depicts rates estimated under a "no-screening" scenario (i.e. a counterfactual baseline). These estimates were obtained by only combining the age and cohort effects (f_A and f_C), previously estimated from the full model, using a 5 weeks/year age adjustment (long-dash dot line) for biological age. We estimated that by 2013, approximately 65 000 cases (48%) of the expected cancers had been prevented since the introduction of screening in 1988. Similarly, using the power link function, the estimated number of prevented cancers was over 55000 (44%). Results showed that, under the no-screening scenario, ASR would have been 3-fold higher in 2013 using the log link; observed ASR 12.8 (95%Cis: 12.3 to 13.3) vs. no-screening scenario 37.9 (95%Cis: 36.4 to 39.3). We also found this will still be the case in 2030 assuming the current scenario continued. In 2013, incidence rates in the no-screening scenario were estimated to be higher across all age groups compared to the observed rates (Table 2). As to be expected, the estimated rates under the no-

For peer review only - http://bmjopen.bmj.com/site/about/guidelines.xhtml

For peer review only

screening scenario were lower when using the power link compared to the log link function; however, both models showed the protective effect of screening on incidence rates.

Table 2. Incidence rates in 2013 by age group. Observed rates vs. estimated rates under the no-screening scenario using a 5-year age adjustment and the log and power link function

	Observed Data	No screening – Log link	No screening – Power link
**20-34**	14.8 (13.8 – 15.9)	34.7 (32.3 – 37.1)	29.1 (27.1 – 31.1)
**35-49**	14.6 (13.6 – 1.6)	49.6 (46.2 – 53.0)	41.0 (38.2 – 43.9)
**50-64**	9.8 (8.9 – 10.7)	37.9 (34.5 – 41.3)	34.5 (31.5 – 37.6)
**65+**	10.2 (9.2 – 11.1)	18.9 (17.1 – 20.7)	18.4 (16.7 - 20.2)

Screening effect

We explored the effect of “screening” (i.e. period effects) across different age groups. Figure 2 shows the relative risks (RRs) associated with the period effect for each age-group plotted over year of diagnosis. The figure shows RRs obtained using chronological age compared to biological age with a 5 weeks/year adjustment. The biological age adjustment does not affect results for women aged 50+ based on the model assumptions. Overall, screening reduces the relative risk associated with cancer incidence. This protective effect is more pronounced in women aged 35-64 (vs. <35) but it can still be observed amongst women aged 65-74 (and even 75-84), although steadily waning, as shown in Figure 2.

FIGURE2

DISCUSSION

Our results showed an AC model could fit incidence rates well for years pre-1988, which indicates the period effect is a proxy for the introduction of screening. Our modified APC model successfully captured past trends and efficiently estimated future incidence rates. Thus, our study corroborates the effectiveness of screening at reducing cervical cancer incidence across all ages. [7-10] Franceschi and Vaccarella estimated screening had prevented more than 30000 cancers between 1983 and 2007 and we estimated approximately 65000 were prevented between 1988 and 2013. This increase in the number of prevented cancer is driven by the fact we have 6 additional years of data. The model showed the period effect, capturing screening, was greater amongst women aged 35-64 (vs. <35), potentially as they have attended several screening rounds. Moreover, it captured the protective effect of screening in older women (65+). Indeed, we observed a waning “protective” effect which is still

For peer review only - http://bmjopen.bmj.com/site/about/guidelines.xhtml

considerable at age 74. This is in line with previous results by Castañon and colleagues [27] based on individual exposure to screening (rather than trends). The data suggest there is an effect of screening among women aged 65-69 in the first years after the introduction of the screening program. This observation is supported by data, which indicate women in this age group were undergoing (opportunistic) screening. [30]

We quantified the effectiveness of the screening programme by comparing observed rates with rates estimated under a no-screening scenario. Results suggest that, had screening not been introduced, overall incidence rates would be 3-fold higher, which is in line with previous results.[31] Overall, our results confirm findings from previous cohort and case-control studies while using population data, which are readily available. [24] In spite of the protective effect of screening, rates of cervical cancer are projected to increase over time, especially amongst women aged 35-64, potentially due to increased incidence of HPV infection and falling screening attendance. [32] These results should, however, be interpreted with caution for two reasons. Firstly, they rely on the assumption that the period effects are due to screening and secondly, we assume that the current scenario will continue. In reality, we are expecting HPV vaccination and the imminent introduction of HPV screening to have a further protective effect on women. [15 32] The counterfactual baseline obtained under the no-screening scenario can be used to quantify the effect of vaccination and HPV screening in the future.

Our results indicate the importance of capturing earlier and prolonged HPV exposure, together with changes in exposure intensity, when estimating future cervical cancer incidence. The assumption that women are exposure to HPV at an earlier age in more recent cohorts is supported by changes in sexual behaviour (e.g. earlier age at first intercourse) captured by data from NATSAL-3. [14] Failing to account for changes in age of first exposure to HPV leads to unrealistic future rates, akin to those observed in countries where screening is not systematically offered. [10] This is particularly noticeable amongst women aged 35-64 rather than younger women (<35). The impact of modelling earlier exposure is minimal amongst women under the age of 35 since these observations dictate the model fit.

In a recent study, predicted cervical cancer rates for 2035 were higher than prior to 1988, especially amongst women aged 35 to 64. [11] These results may be due to the use of a standard APC model, which only captures increased intensity of exposure that is expected to last throughout a woman's lifetime. Moreover, we noticed the standard APC model failed to capture the differential effect of screening by age group. Indeed, using chronological age in our data, screening appeared to have a greater protective effect amongst younger women (<35) than those aged 35-64. This is counter to previous evidence which found screening to be more effective in older women (40+ vs. <40).[23-26] These observations further support the need to consider cohort effects that account for changes in age

For peer review only - http://bmjopen.bmj.com/site/about/guidelines.xhtml

at first exposure in addition to changes in the proportion of the population exposed, already captured by conventional APC models.

The main strength of our analysis is the use of data broken down by single year of age and year of diagnosis over a 40-year period which permit predictions using modelling; however, there are some limitations. A typical limitation of APC analysis is that predicted rates are dependent on the model's assumptions, but our validation analyses suggest our assumptions are valid under the current scenario. Additionally, our study does not model the effect of HPV vaccination on future incidence rates in younger cohorts. In the UK, HPV vaccination of girls aged 12 to 13 years started in 2008 and a catch-up programme ran for women up to the age of 18 for the following 2-3 years. Vaccination uptake has been estimated to be around 80% amongst eligible girls. [33] However, the counterfactual no-screening scenario baseline from our model, was used in a microsimulation study to evaluate the appropriate screening intensity in HPV vaccinated and unvaccinated women. [16] This counterfactual baseline can also be used as a reference to quantify the effect of HPV vaccination and screening in the future. We interpret the reduction in incidence rates being driven by the introduction of the screening programme as indicated by decreasing relative risks of the period effect. It is, nonetheless, possible that the period effect is capturing other factors; however, such period reductions are not observed in Eastern European countries where screening is not adequately implemented and, therefore, we believe the reduction is driven by screening in line with previous work. [7 34] Our incidence rates are calculated for the overall population and we did not exclude women who have undergone hysterectomy from the denominator. However, in England and Wales hysterectomy prevalence is low, with a rate of 10.3% amongst women aged 25-64 (1991-995), and the extrapolated hysterectomy prevalence appears stable up to 2030. [35] Therefore, we are confident our estimated trends closely mirror the trends in cancer incidence in women with a cervix, even though, we acknowledge, the rates in older women with a cervix will be somewhat higher than the rates in all older women.

Conclusions

Using a modified APC model on cancer registry data, we confirmed the protective effect of screening with this effect lasting for over 10 years after the last screening invitation. Our results support the epidemiology of cervical cancer in younger cohorts being affected by earlier onset of sexual behaviour, as reported in previous studies, [14 28] and not just the greater number of sexual partners. We found that failing to account for changes in age of first HPV exposure leads to unrealistic future estimates and, therefore, our modified approach should be used when modelling incidence of cervical cancer. Moreover, the use of our model is not restricted to cervical cancer but could be implemented by researchers studying other diseases (e.g. lung cancer) where age at exposure, as well as intensity of exposure, may determine future age-specific incidence.

For peer review only - http://bmjopen.bmj.com/site/about/guidelines.xhtml

For peer review only

For peer review only - http://bmjopen.bmj.com/site/about/guidelines.xhtml

REFERENCES

1. Andrae B, Kemetli L, Sparen P, et al. Screening-preventable cervical cancer risks: evidence from a nationwide audit in Sweden. *Journal of the National Cancer Institute* 2008;100(9):622-9. doi: 10.1093/jnci/djn099 [published Online First: 2008/05/01]
2. IARC Working Group on Cervical Cancer Screening. Efficacy of Screening chapter. In: Hakama M MA, Day NE, eds, ed. Screening for Cancer of the Uterine Cervix IARC Scientific Publications N 76. Lyon: IARC1986.
3. Lowy DR. HPV vaccination to prevent cervical cancer and other HPV-associated disease: from basic science to effective interventions. *The Journal of Clinical Investigation* 2016;126(1):5-11. doi: 10.1172/JCI85446
4. Foley G, Alston R, Geraci M, et al. Increasing rates of cervical cancer in young women in England: an analysis of national data 1982-2006. *Br J Cancer* 2011;105(1):177-84. doi: 10.1038/bjc.2011.196 [published Online First: 2011/06/10]
5. Cuzick J, Sasieni P, Davies P, et al. A systematic review of the role of human papilloma virus (HPV) testing within a cervical screening programme: summary and conclusions. *Br J Cancer* 2000;83(5):561-5. doi: 10.1054/bjoc.2000.1375 [published Online First: 2000/08/17]
6. Walboomers JM, Jacobs MV, Manos MM, et al. Human papillomavirus is a necessary cause of invasive cervical cancer worldwide. *The Journal of pathology* 1999;189(1):12-9. doi: 10.1002/(sici)1096-9896(199909)189:1<12::aid-path431>3.0.co;2-f [published Online First: 1999/08/19]
7. Franceschi S, Vaccarella S. Beral's 1974 paper: A step towards universal prevention of cervical cancer. *Cancer Epidemiology* 2015;39(6):1152-56. doi: http://dx.doi.org/10.1016/j.canep.2015.10.019
8. Sasieni P, Adams J. Analysis of cervical cancer mortality and incidence data from England and Wales: evidence of a beneficial effect of screening. *Journal of the Royal Statistical Society: Series A (Statistics in Society)* 2000;163(2):191-209. doi: 10.1111/1467-985X.00165
9. Vaccarella S, Franceschi S, Engholm G, et al. 50 years of screening in the Nordic countries: quantifying the effects on cervical cancer incidence. *Br J Cancer* 2014;111(5):965-69. doi: 10.1038/bjc.2014.362
10. Vaccarella S, Franceschi S, Zaridze D, et al. Preventable fractions of cervical cancer via effective screening in six Baltic, central, and eastern European countries 2017-40: a population-based study. *The Lancet Oncology* 2016;17(10):1445-52. doi: 10.1016/s1470-2045(16)30275-3 [published Online First: 2016/08/28]
11. Smittenaar CR, Petersen KA, Stewart K, et al. Cancer incidence and mortality projections in the UK until 2035. *Br J Cancer* 2016;115(9):1147-55. doi: 10.1038/bjc.2016.304 [published Online First: 2016/10/26]
12. Plummer M, Peto J, Franceschi S. Time since first sexual intercourse and the risk of cervical cancer. *International journal of cancer* 2012;130(11):2638-44. doi: 10.1002/ijc.26250 [published Online First: 2011/06/28]
13. Cuzick J, Sasieni P, Singer A. Risk factors for invasive cervix cancer in young women. *European journal of cancer (Oxford, England : 1990)* 1996;32a(5):836-41. [published Online First: 1996/05/01]
14. Mercer CH, Tanton C, Prah P, et al. Changes in sexual attitudes and lifestyles in Britain through the life course and over time: findings from the National Surveys of Sexual Attitudes and Lifestyles (Natsal). *Lancet (London, England)* 2013;382(9907):1781-94. doi: 10.1016/s0140-6736(13)62035-8 [published Online First: 2013/11/30]
15. Castanon A, Landy R, Sasieni P. By how much could screening by primary human papillomavirus testing reduce cervical cancer incidence in England? *Journal of medical screening* 2017;24(2):110-12. doi: 10.1177/0969141316654197 [published Online First: 2016/07/02]

For peer review only

For peer review only - http://bmjopen.bmj.com/site/about/guidelines.xhtml

16. Landy R, Windridge P, Gillman MS, et al. What cervical screening is appropriate for women who have been vaccinated against high risk HPV? A simulation study. *International journal of cancer* 2018;142(4):709-18. doi: 10.1002/ijc.31094 [published Online First: 2017/10/13]
17. ONS. Mid-1971 to Mid-2013 Population Estimates for UK, constituent countries and English regions, 2014.
18. Castanon A, Leung VMW, Landy R, et al. Characteristics and screening history of women diagnosed with cervical cancer aged 20-29 years. *Br J Cancer* 2013;109(1):35-41. doi: 10.1038/bjc.2013.322
19. Castanon A, Sasieni P. Is the recent increase in cervical cancer in women aged 20-24years in England a cause for concern? *Preventive medicine* 2018;107:21-28. doi: 10.1016/j.ypmed.2017.12.002 [published Online First: 2017/12/17]
20. Lancucki L, Sasieni P, Patnick J, et al. The impact of Jade Goody's diagnosis and death on the NHS Cervical Screening Programme. *Journal of medical screening* 2012;19(2):89-93. doi: 10.1258/jms.2012.012028 [published Online First: 2012/06/02]
21. Sasieni P, Castanon A. Dramatic increase in cervical cancer registrations in young women in 2009 in England unlikely to be due to the new policy not to screen women aged 20-24. *Journal of medical screening* 2012;19(3):127-32. doi: 10.1258/jms.2012.012081 [published Online First: 2012/10/25]
22. Moller B, Fekjaer H, Hakulinen T, et al. Prediction of cancer incidence in the Nordic countries: empirical comparison of different approaches. *Stat Med* 2003;22(17):2751-66. doi: 10.1002/sim.1481 [published Online First: 2003/08/27]
23. Sasieni P, Adams J, Cuzick J. Benefit of cervical screening at different ages: evidence from the UK audit of screening histories. *Br J Cancer* 2003;89(1):88-93. doi: 10.1038/sj.bjc.6600974 [published Online First: 2003/07/03]
24. Sasieni P, Castanon A, Cuzick J. Effectiveness of cervical screening with age: population based case-control study of prospectively recorded data. *BMJ (Clinical research ed)* 2009;339:b2968. doi: 10.1136/bmj.b2968 [published Online First: 2009/07/30]
25. Zappa M, Visioli CB, Ciatto S, et al. Lower protection of cytological screening for adenocarcinomas and shorter protection for younger women: the results of a case-control study in Florence. *Br J Cancer* 2004;90(9):1784-6. doi: 10.1038/sj.bjc.6601754 [published Online First: 2004/05/20]
26. Lonnberg S, Anttila A, Luostarinen T, et al. Age-specific effectiveness of the Finnish cervical cancer screening programme. *Cancer epidemiology, biomarkers & prevention : a publication of the American Association for Cancer Research, cosponsored by the American Society of Preventive Oncology* 2012;21(8):1354-61. doi: 10.1158/1055-9965.epi-12-0162 [published Online First: 2012/06/06]
27. Castanon A, Landy R, Cuzick J, et al. Cervical screening at age 50-64 years and the risk of cervical cancer at age 65 years and older: population-based case control study. *PLoS medicine* 2014;11(1):e1001585. doi: 10.1371/journal.pmed.1001585 [published Online First: 2014/01/24]
28. Simms I, Fairley CK. Epidemiology of genital warts in England and Wales: 1971 to 1994. *Genitourinary medicine* 1997;73(5):365-7. [published Online First: 1998/04/16]
29. Sasieni P. Age-period-cohort models in Stata. *Stata Journal* 2012;12(1):45-60.
30. DoH. Cervical Cytology: Summary Information From Form KC53. London, United Kingdom: Department of Health Statistics Division, 1989-90.
31. Landy R, Pesola F, Castanon A, et al. Impact of cervical screening on cervical cancer mortality: estimation using stage-specific results from a nested case-control study. *Br J Cancer* 2016;115(9):1140-46. doi: 10.1038/bjc.2016.290 [published Online First: 2016/10/26]
32. Castanon A, Landy R, Pesola F, et al. Prediction of cervical cancer incidence in England, UK, up to 2040, under four scenarios: a modelling study. *The Lancet Public health* 2018;3(1):e34-e43. doi: 10.1016/s2468-2667(17)30222-0 [published Online First: 2018/01/09]

For peer review only - http://bmjopen.bmj.com/site/about/guidelines.xhtml

33. Tiley K, White J. Human Papillomavirus (HPV) vaccination coverage in adolescent females in England: 2016/17. http://www.webcitation.org/73HhxGD0g, 2017.
34. Vaccarella S, Lortet-Tieulent J, Plummer M, et al. Worldwide trends in cervical cancer incidence: impact of screening against changes in disease risk factors. *European journal of cancer (Oxford, England : 1990)* 2013;49(15):3262-73. doi: 10.1016/j.ejca.2013.04.024 [published Online First: 2013/06/12]
35. Redburn JC, Murphy MF. Hysterectomy prevalence and adjusted cervical and uterine cancer rates in England and Wales. *BJOG : an international journal of obstetrics and gynaecology* 2001;108(4):388-95. [published Online First: 2001/04/18]

For peer review only

For peer review only - http://bmjopen.bmj.com/site/about/guidelines.xhtml

CAPTIONS

Figure 1. Trends and projections based on our modified APC model (using the log link function) across different age groups. The solid circles represent the observed data. Estimated rates for the observed period are for chronological age (short-dash dot) and biological age using a 5 week/year adjustment (solid line). The greyed area shows the potential range of estimated future rates based on the chronological age using 2 and 7 week/year age adjustments (biological age). The rates estimated under the no-screening scenario are represented by the long-dash dot line.

Figure 2. Relative Risk (RR) of the period effect by age group for a) chronological age (solid circle), and b) biological age using a 5 weeks/year age adjustment (hollow circle).

For peer review only

For peer review only - http://bmjopen.bmj.com/site/about/guidelines.xhtml

Funding: The research was supported by a grant from Cancer Research UK, C8162/A16892.

Competing interests: None

Authors' contributions: FP was responsible for study design, data analysis and drafted the paper. PS was responsible for study concept and design and contributed to editing of the paper.

Data sharing statement: The data are available and can be obtained from Public Health England through the Office for Data Release (ODR). The authors can provide data specification for the data request

For peer review only

For peer review only - http://bmjopen.bmj.com/site/about/guidelines.xhtml

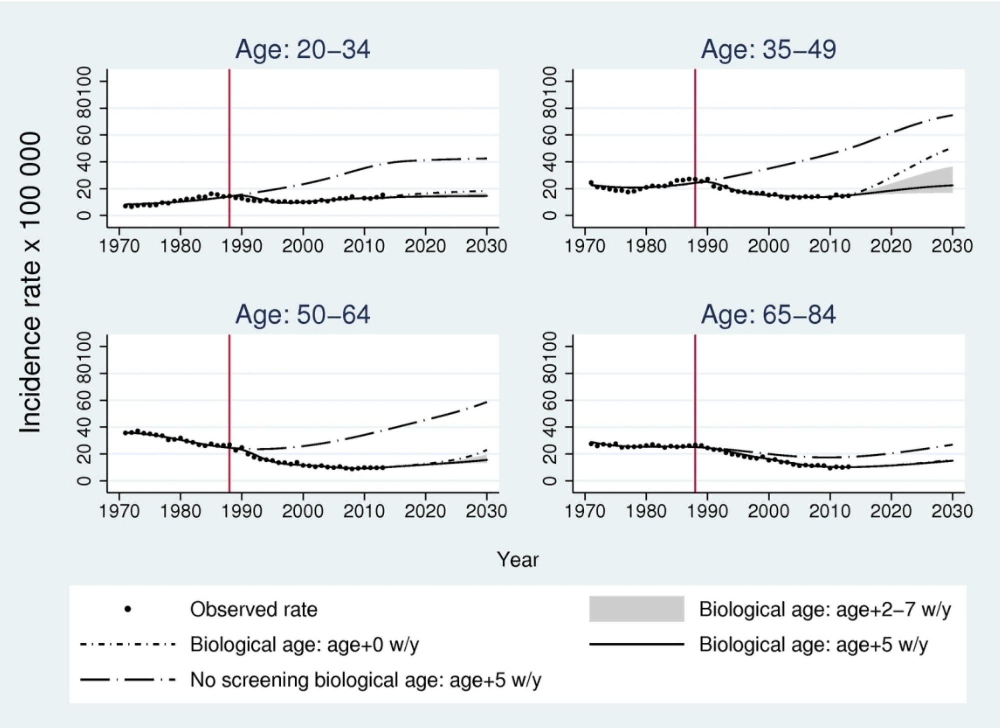

Figure 1. Trends and projections based on our modified APC model (using the log link function) across different age groups. The solid circles represent the observed data. Estimated rates for the observed period are for chronological age (short-dash dot) and biological age using a 5 week/year adjustment (solid line). The greyed area shows the potential range of estimated future rates based on the chronological age using 2 and 7 week/year age adjustments (biological age). The rates estimated under the no-screening scenario are represented by the long-dash dot line.

139x101mm (300 x 300 DPI)

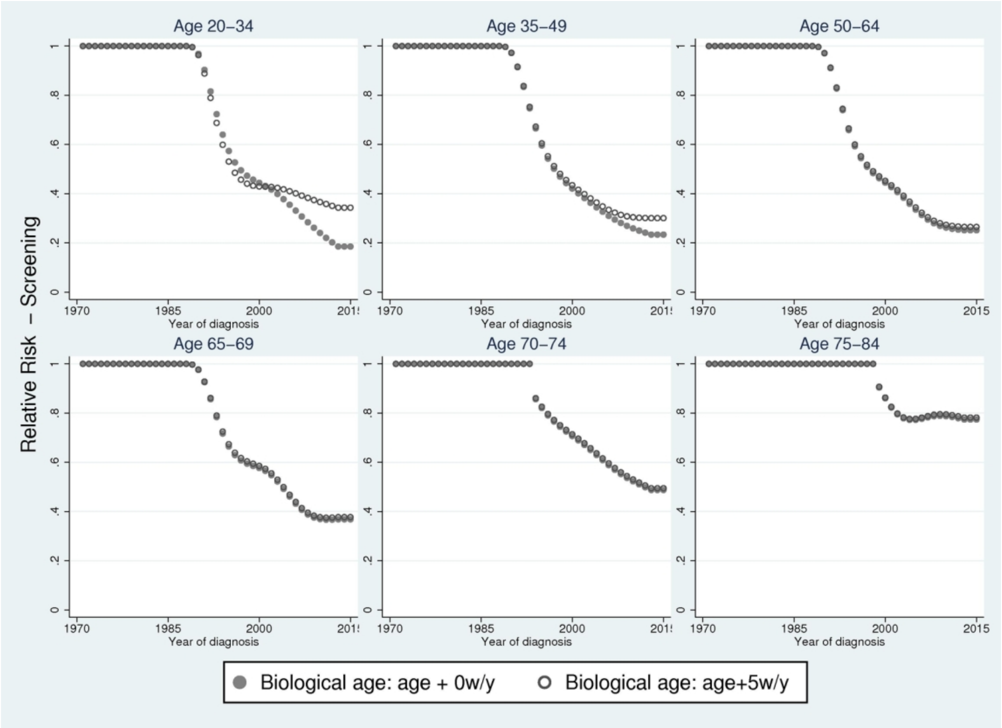

Figure 2. Relative Risk (RR) of the period effect showing the effect of screening by age group for a) chronological age (solid circle), and b) biological age using a 5-week/year age adjustment (hollow circle).

139x101mm (300 x 300 DPI)

Supplementary materials for "The impact of screening on cervical cancer incidence in England: a time trend analysis"

Supplementary 1

Supplementary Table 1. Model fit for data 1971 to 1987 using the log link function

		Log link		Power Link	
	df	AIC	Pseudo R^2	AIC	Pseudo R2
Null		323.4	0	323.4	0
A	1063	22.9	.929	22.9	.929
Adrift (in cohort)	1062	22.8	.930	22.7	.930
AP	1060	22.1	0.932	21.9	.932
AC	1059	14.4	0.956	14.9	.954
APC	1056	14.2	0.956	14.6	.955
ACP*A	1041	11.9	0.963	12.3	.962

For peer review only

Supplementary 2

Validation analysis and HPV age adjustment selection

In our validation analyses to identify the best weeks/year adjustment to define biological age, we used observed data between 1971 and 2001 to predict incidence rates up to 2013 applying a number of weeks/year adjustments (i.e. x= 0 to 12) in our modified APC model. To quantify how well the model predicted incidence for years 2002 to 2013, we used a modified Pearson Chi-squared statistic:

$$(estimated-observed)^2/(0.5+observed) \qquad (1)$$

where the observed value was used in the denominator as it is the best estimator of the variance of the observed count since, for projections, the 'expected count' may not reflect the true expectation. Small values indicate a good fit to the data.

Figure 1 plots the obtained Pearson's Chi-squared against the various age adjustments. Our validation analyses showed that, overall, a 2- to 7-weeks/year adjustment offered a good fit to the data and greater adjustments (i.e. 5-7 weeks/year) fit the data best when projections were longer into the future (i.e. 6+ years).

For peer review only

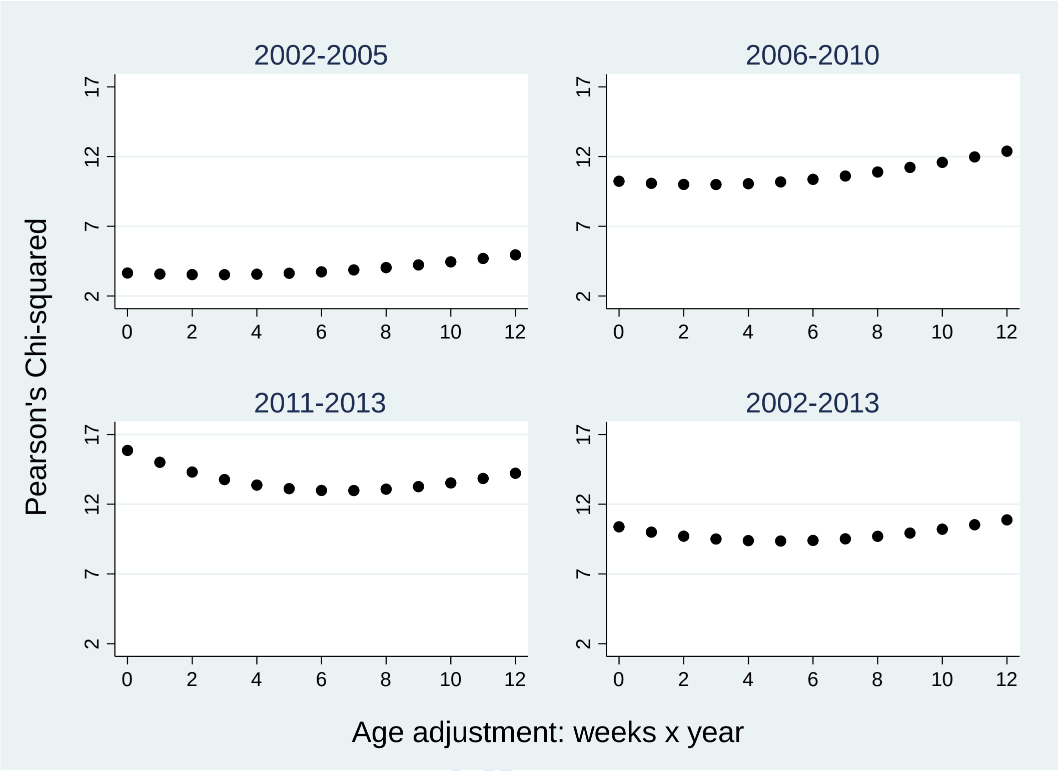

Figure S2. Goodness of fit indices for a number of weeks/year age adjustments (i.e. biological age) using observed data between 1971 and 2001 to predict incidence rates up to 2013.

Supplementary 3

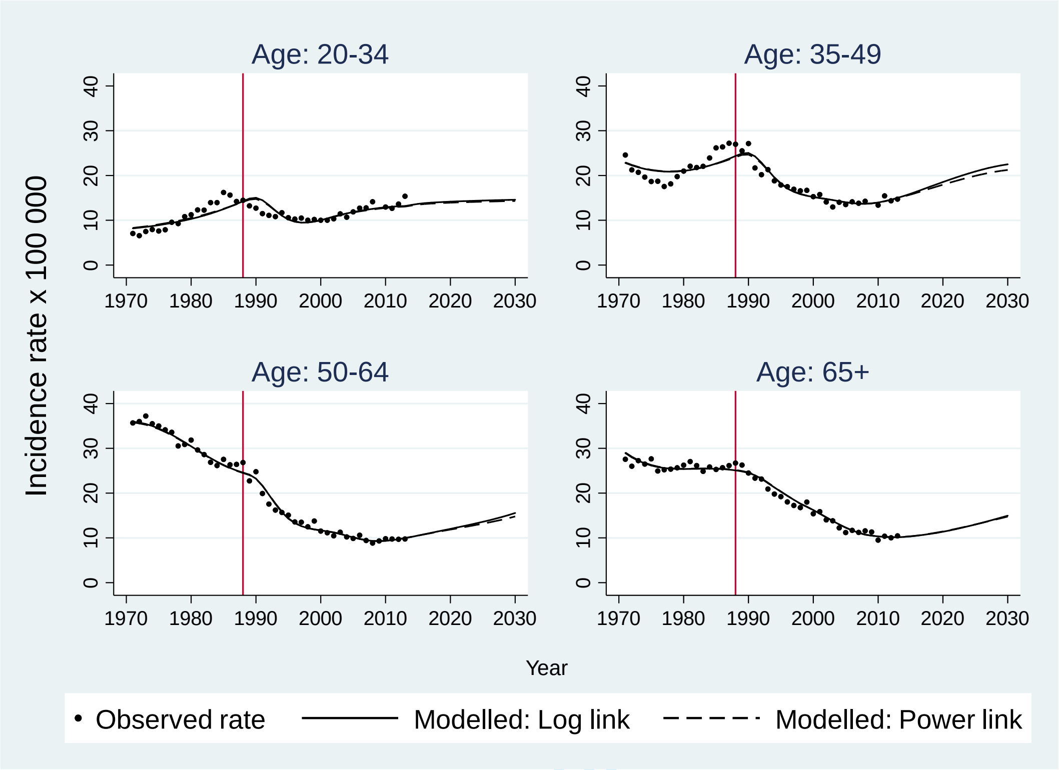

Figure S1. Cervical cancer incidence by age: Observed and estimated rates, using the log and 'power-5' link functions and a 5 weeks/year age adjustment (i.e. biological age)

Supplementary 4

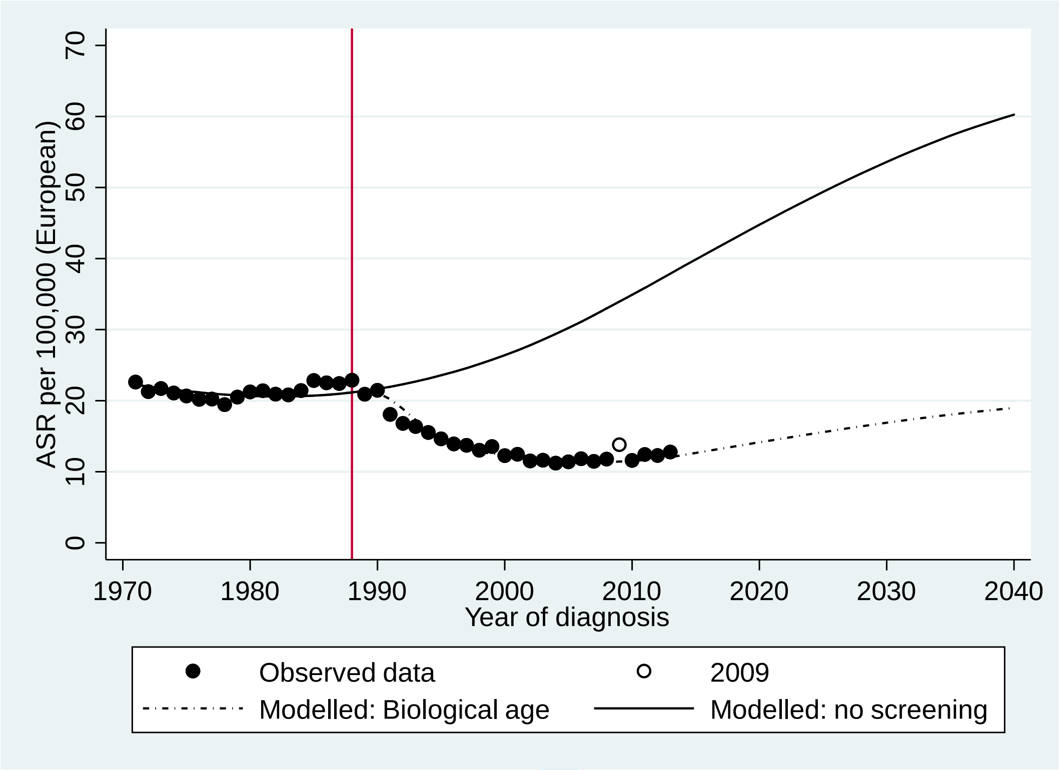

Figure S3. Age Standardised Rate (European population) in women aged 20 to 84. Cervical cancer incidence comparing observed data, projections under the current scenario and the no-screening scenario using a 5 weeks/year age adjustment (i.e. biological age).

For peer review only
